# SIB1‐SEC23A undergo ER to chloroplast relocalization to mediate immunity in *Arabidopsis thaliana*

**DOI:** 10.1111/jipb.70251

**Published:** 2026-04-12

**Authors:** Jialin Peng, Huan Zhong, Jianan Zhang, Hailei Zhang, Wuzhen Liu, Yonglun Zeng, Liwen Jiang, Yiji Xia

**Affiliations:** ^1^ Department of Biology Hong Kong Baptist University Hong Kong 999077 China; ^2^ College of Biological and Environmental Sciences Zhejiang Wanli University Ningbo 315100 China; ^3^ State Key Laboratory of Plant Diversity and Specialty Crops and Guangdong Provincial Key Laboratory of Applied Botany South China Botanical Garden, Chinese Academy of Sciences Guangzhou 510650 China; ^4^ University of Chinese Academy of Sciences Beijing 100193 China; ^5^ School of Life Sciences, Centre for Cell & Developmental Biology, AoE Centre for Organelle Biogenesis and Function The Chinese University of Hong Kong Hong Kong 999077 China; ^6^ State Key Laboratory of Agrobiotechnology The Chinese University of Hong Kong Hong Kong 999077 China; ^7^ Institute of Plant Molecular Biology and Agricultural Biotechnology The Chinese University of Hong Kong Hong Kong 999077 China; ^8^ AoE Centre for Plant Vacuole Biology and Biotechnology The Chinese University of Hong Kong Hong Kong 999077 China

**Keywords:** Arabidopsis, chloroplast, ER stress, inter‐organelle communication, plant immunity, SEC23A, SIB1

## Abstract

Inter‐organellar communication has emerged as a critical factor in maintaining cellular homeostasis under stress conditions. Chloroplasts function not only as central organelles for energy production but are also increasingly recognized as stress sensors and signal integrators. SIGMA FACTOR‐BINDING PROTEIN 1 (SIB1), encoded by a nuclear gene, has been identified as a positive regulator of plant immunity, with localization in both chloroplasts and the nucleus. In this study, we identified Arabidopsis SEC23A, a COPII complex component known to mediate membrane trafficking between the endoplasmic reticulum (ER) and the Golgi apparatus, as a novel interactor of SIB1. Our findings reveal that under normal conditions, both SIB1 and SEC23A localize to the ER, while SIB1 is also localized in the nucleus. Upon ER stress and/or treatment with the immunity inducer salicylate, both proteins are relocated from the ER to the chloroplasts. Notably, SEC23A, similar to SIB1, also functions as a positive regulator of disease resistance. In response to pathogen infection, *SIB1* and *SEC23A* downregulate expression of chloroplast‐ and nucleus‐encoded genes associated with photosynthesis while enhancing expression of defense‐related genes. Together, our findings reveal a previously uncharacterized pathway of ER–chloroplast communication mediated by SIB1 and SEC23A during plant stress responses and immunity, providing novel insights into the intricate regulatory networks that govern inter‐organellar communication under stress in plants.

## Graphical abstract

SEC23A mediates membrane trafficking between the endoplasmic reticulum (ER) and Golgi apparatus and interacts with the immunity protein SIGMA FACTOR‐BINDING PROTEIN1 in Arabidopsis. Under stress, both proteins relocate from the ER to chloroplasts, suppressing photosynthesis genes and enhancing defense genes.

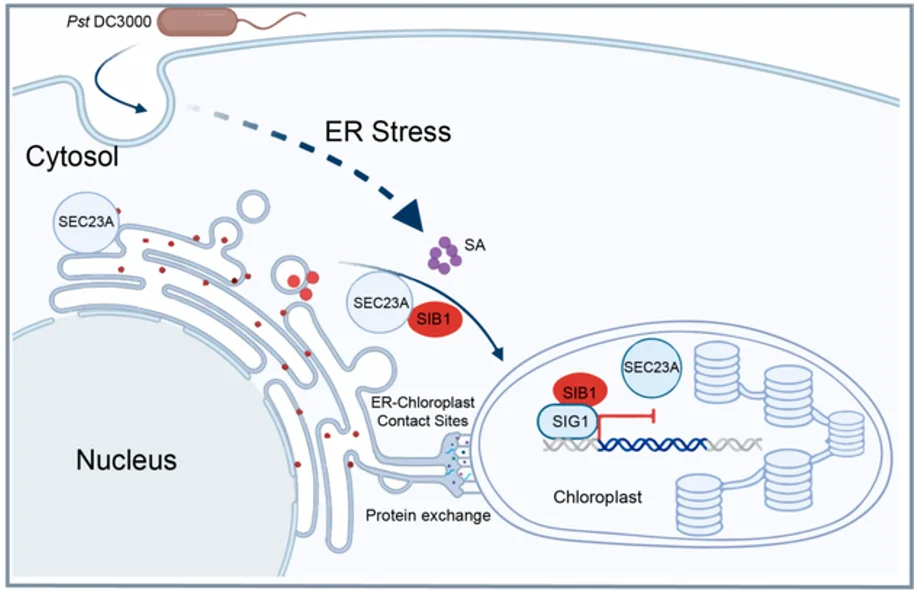

## INTRODUCTION

Plants have developed complex tolerance mechanisms that combine different stress signals to regulate growth and development in response to ever‐changing environments (Nakashima et al., 2009). Inter‐organellar communication has been widely documented as a critical mechanism in preserving cellular homeostasis during environmental stress. It is also important for successful innate immune responses that confer defense against pathogens (Nomura et al., 2012; Hanson and Hines, 2018).

Chloroplasts, originating from a photosynthetic bacterial endosymbiont (Hirosawa et al., 2021), are emerging as hubs for the integration of diverse environmental stimuli and determinants of downstream responses (de Torres Zabala et al., 2015; Zhao et al., 2016; Zhu, 2016; de Souza et al., 2017). Photosynthesis is now believed to be strongly connected with the immune response. Activation of the defense mechanism consumes high amounts of energy; thus, increasing photosynthetic production could be beneficial. On the other hand, increased photosynthesis could increase the availability of carbon compounds for the invading pathogen, which might facilitate infection (Serrano et al., 2016). Chloroplasts are known to play a role in the plant defense response through different modes of action, including as the sources of calcium and reactive oxygen species (ROS) bursts, and biosynthesis of salicylic acid (SA) (Chan et al., 2016; Medina‐Puche et al., 2020). Several effector proteins secreted by pathogens into host plants to suppress the host defense response are targeted at chloroplasts (Jelenska et al., 2007; Rodríguez‐Herva et al., 2012; Li et al., 2014; Xu et al., 2019). Studies have shown that pathogen infection can induce the formation of stromules—stroma‐filled tubules extending from various plastids, including proplastids and chloroplasts. These structures may physically connect to the nucleus, facilitating inter‐organellar transport of proteins and signaling molecules such as H_2_O_2_ (Caplan et al., 2015).

The endoplasmic reticulum (ER) also plays a critical role in the stress response in addition to its central role in vesicle trafficking and protein glycosylation. Both exogenous and endogenous conditions, such as environmental stresses, pathogens, drought, and salinity, increase the demand for the protein folding machinery that secretes proteins into the ER (Bao and Howell, 2017; Nawkar et al., 2018). This increased demand can cause an accumulation of unfolded or misfolded proteins, triggering the unfolded protein response (UPR) (Iwata and Koizumi, 2012; Liu and Li, 2019; Ko and Brandizzi, 2024). The UPR in plants operates through two conserved pathways: The IRE1–bZIP60 branch, where IRE1‐mediated splicing generates a truncated bZIP60 transcription factor that translocates to the nucleus, and the bZIP17/28 branch, where ER stress triggers Golgi‐mediated proteolytic cleavage of membrane‐anchored bZIP17/28, releasing their cytosolic domains for nuclear translocation and stress‐responsive gene regulation (Howell, 2021). ER–chloroplast contact sites maintain lipid homeostasis through specialized protein systems, including trigalactosyldiacylglycerol (TGD) family ABC transporters that shuttle lipids from the ER to thylakoids (Fan et al., 2015) and membrane‐bound enzymes like phosphatidylcholine (PC) synthase and CLIP1 lipase/acylhydrolase that directly modify interfacial lipids (Mehrshahi et al., 2013). These dynamic interactions support both basal metabolism and stress adaptation in plants (Hurlock et al., 2014).

Membrane vesicle trafficking plays a central role in transporting proteins and other molecules between different membrane‐enclosed compartments. Coat Protein Complex II (COPII) is indispensable for membrane trafficking from the ER to the Golgi. In eukaryotes, the COPII complex consists of five evolutionarily conserved cytosolic components, including Sar1, Sec23, Sec24, Sec13, and Sec31 (Lord et al., 2013). Among the COPII core components, Sec23 acts as a Sar1 GTPase activator and Sec24 acts as a cargo receptor for protein sorting (Zanetti et al., 2012; Miller and Schekman, 2013). Arabidopsis shows expanded COPII diversity, including seven Sec23 paralogs with specialized functions in ER export and development (Chung et al., 2016). AtSEC23A/D jointly regulate pollen wall formation (Aboulela et al., 2018), and the interaction between AtSEC23A and MTM2 modulates autophagy at ER exit sites (Li et al., 2025).

In prokaryotic cells, a sigma factor recognizes the promoter sequence and recruits the core RNA polymerase to initiate transcription (Gruber and Gross, 2003). *Escherichia coli* possesses seven sigma factors, some of which regulate the transcription of housekeeping genes, while others control genes are involved in specific pathways. The activity of sigma factors can be suppressed by anti‐sigma factor proteins (Trevino‐Quintanilla et al., 2013; Feklistov et al., 2014). Since plastids are derived from once free‐living bacterial cells, plant sigma factors function in plastids to determine the promoter specificity of the major plastid RNA polymerase and thus regulate plastome gene expression. Chloroplast transcription in higher plants is mediated by two RNA polymerases: the plastid‐encoded PEP (a eubacterial‐type multisubunit enzyme) and the nuclear‐encoded NEP (a T_7_ phage‐type single‐subunit enzyme). While PEP—regulated by nuclear‐encoded sigma subunits—dominates chloroplast biogenesis and maintenance, NEP can also transcribe many plastid genes, demonstrating functional overlap between the two systems (Kanamaru and Tanaka, 2004). There are six nuclear‐encoded sigma factors (SIG1/2/3/4/5/6) in Arabidopsis that likely act in PEP‐mediated transcription in plastids (Tanaka et al., 1997). Morikawa et al. identified the protein SIGMA FACTOR‐BINDING PROTEIN 1 (SIB1) (At3g56710) as one that binds to Sigma Factor 1 (also named SigA) of Arabidopsis (Morikawa et al., 2002). SIB1 is encoded by a nuclear gene and imported into chloroplasts after its synthesis in the cytosol. SIB1 was identified as a positive regulator of plant immunity and was found to be highly induced in response to the infection by the bacterial pathogen *Pseudomonas syringae* (Xie et al., 2010). Later, SIB1 was also found to be localized in the nucleus, where it binds to and acts as a co‐activator of the transcription factor WRKY33, a positive regulator of defense gene expression (Lai et al., 2011). SIB1 was also found to interact with WRKY75 (Zhang et al., 2022) and WRKY57 (Jiang and Yu, 2016). Besides, SIB1 and its homolog SIB2 were found to play a role in abscisic acid‐mediated leaf senescence and seed germination by inhibiting WRKY75 function in the nucleus (Zhang et al., 2022). SIB1 was reported to undergo co‐translational N‐terminal acetylation and posttranslational ubiquitination to influence its stability and the nuSIB1‐mediated stress responses (Li et al., 2020). SIB1 differentially regulates chloroplast‐ and nucleus‐encoded photosynthetic genes, inducing photosystem II photoinhibition and singlet oxygen (^1^O_2_) accumulation. This activates EX1‐mediated retrograde signaling to trigger programmed cell death (Lv et al., 2019). By interacting with GLK1/2, SIB1 enhances PhANGs expression and ^1^O_2_ production, while its antagonism with LSD1 maintains oxidative homeostasis during stress responses (Li et al., 2022).

Initially, through a yeast two‐hybrid screening using SIB1 as the bait, we identified over a dozen SIB1 interactors, including the previously known interactors SigA and WRKY proteins. Notably, SEC23A, which is involved in membrane trafficking from the ER to the Golgi, was also identified as a SIB1 interactor. This finding prompted us to investigate the role of SEC23A in plant immunity and to determine whether SIB1 is associated with the ER. Our study revealed that SIB1 and SEC23A are associated with the ER under normal conditions but relocate to chloroplasts under ER stress or following treatment with the immune response inducer salicylate. Their normal function appears to involve the suppression of photosynthetic gene expression in both chloroplast and nuclear genomes while enhancing the expression of defense‐related genes. These findings provide novel insights into the ER–chloroplast interaction during stress and immune responses in plants.

## RESULTS

### SIB1 interacts with SEC23A

Our previous study identified SIB1 as a positive regulator of the immune response and one of the genes that showed strong and early induction during infection by *P. syringae* (*Pst*) (Xie et al., 2010). SIB1 was later found to interact with several WRKY proteins in the nucleus to regulate the expression of defense‐related genes (Lai et al., 2011; Jiang and Yu, 2016; Zhang et al., 2022). The multi‐functional nature of SIB1 prompted us to determine if it might have additional functions through interaction with other proteins. Toward this goal, a yeast two‐hybrid screen was conducted to identify additional SIB1‐interacting proteins.

The full‐length SIB1 protein was found to have a transcription‐activating activity and can activate reporter genes when fused with the DNA binding domain used in the Y2H system in yeast cells. Therefore, the full‐length SIB1 is not a suitable bait for Y2H screening. SIB1 consists of 151 amino acids, with residues 1–14 predicted to be a chloroplast signal peptide, residues 16–23 predicted to be a nuclear targeting signal, and residues 58–67 forming the VQ motif (Xie et al., 2010; Lai et al., 2011). The VQ motif is characterized by the consensus sequence FXhVQChTG, with *X* indicating any amino acid and *h* representing a hydrophobic residue (Xie et al., 2010), and this motif has been reported to interact with various WRKY proteins (Xie et al., 2010; Chi et al., 2013). As illustrated in Figure S1A, we created two truncated SIB1 segments, one spanning amino acids 1–100 and the other spanning amino acids 40–151, to test their ability to self‐activate the reporter when fused with a DNA binding domain in Y2H. The SIB1's 1–100 aa (amino acid) fragment showed no ability to activate the reporter, whereas the 40–151 aa fragment retained its activation ability (Figure S1B). Therefore, the 1–100 aa N‐terminus of SIB1 (SIB1‐N) was used as a bait in the yeast two‐hybrid screen for SIB1‐interacting proteins in an Arabidopsis two‐hybrid cDNA library derived from rosette leaves infected with *P. syringae*.

After screening nearly 81 million transformants, 266 positive clones were obtained, and the cDNA inserts were sequenced to identify a total of fifty‐four different genes. Twenty‐seven of the putative interactors with high confidence levels have been identified (Table S1). Among them are SigA, WRKY33, and WRKY25, which have been previously reported to be SIB1 interactors. Two of the transformants contained a sequence from At4g01810, which encodes SEC23A. The interaction between SIB1 and SEC23A was further confirmed using targeted Y2H assays. These tests revealed that their interaction involves the 190–421 amino acid region of SEC23A, which includes the zinc‐finger domain and a half of the Trunk domain (Figure S2A). A truncated SEC23A protein containing amino acids 190–515 or the whole SEC23A protein showed weaker interactions than the 190–421 region (Figure S2B), suggesting that the presence of amino acids 422–515 might affect its interaction with SIB1. To further confirm the interaction surface of the SEC23A–SIB1 complex structure, we have performed structure prediction using AlphaFold3, which suggests that their interaction interface involves the VQ motif of SIB1 and the partial Trunk domain of SEC23A (Figure S2C).

**Figure 1:**
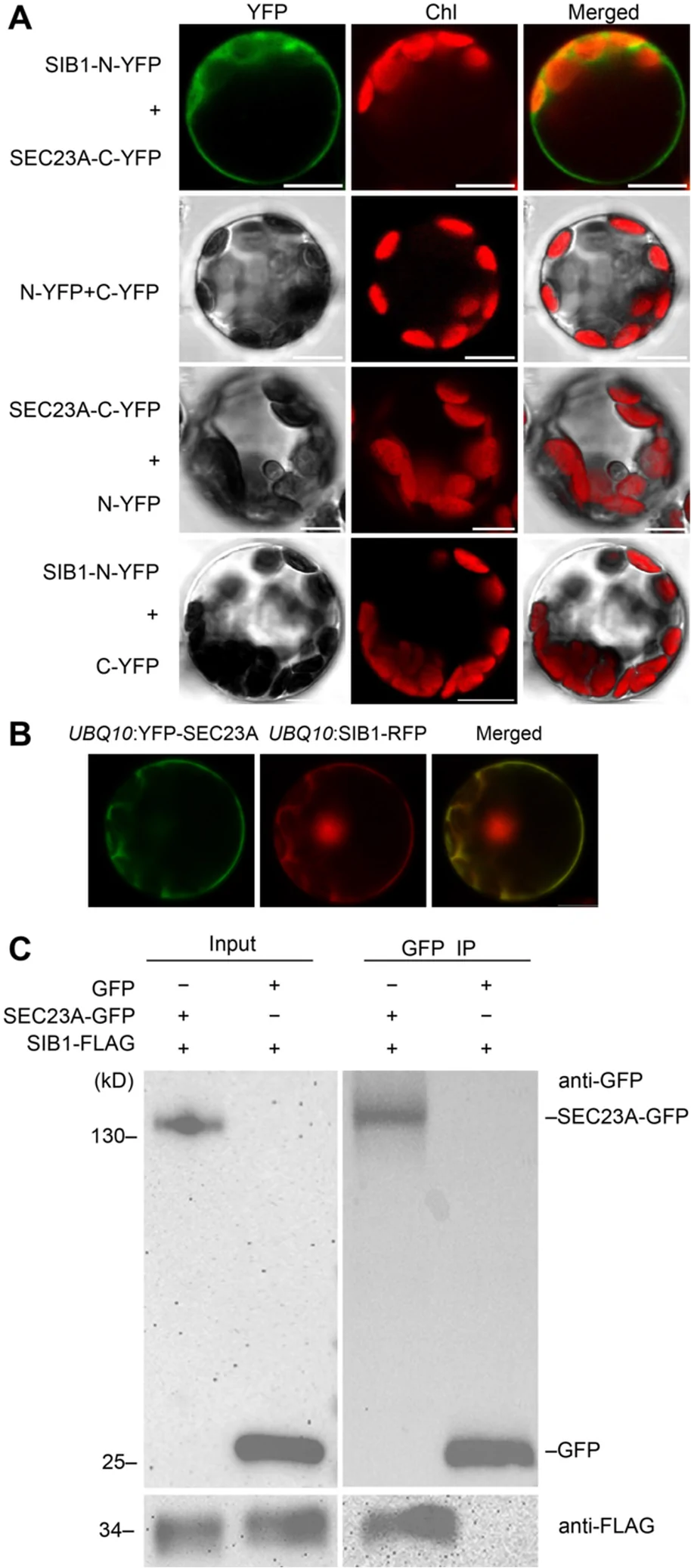
SIB1 interacts with SEC23A **(A)** Bimolecular fluorescence complementation (BiFC) assay showing *in planta* interaction. nYFP and cYFP are the constructs with the N‐terminal and C‐terminal YFP, respectively. The YFP signal was detected in the cytoplasm only when SIB1‐nYFP and SEC23A‐cYFP were co‐expressed. Chl: Chloroplasts. **(B)** YFP‐SEC23A and SIB1‐RFP under the *UBQ10* promoter co‐expressed in Arabidopsis protoplasts under normal conditions. Scale bar = 10 μm. **(C)** Leaf tissues from *Nicotiana benthamiana* plants expressing both *UBQ10*:SIB1‐FLAG and either *35S*:GFP or *35S:*SEC23A‐GFP were used for protein extraction. Protein complexes were immunoprecipitated with GFP‐trap agarose beads, followed by immunoblot analysis with both anti‐GFP and anti‐FLAG antibodies. The experiments were repeated three times, with similar results.

Bimolecular fluorescence complementation (BiFC) assays were first used to determine if SIB1 and SEC23A interact *in planta*. When SIB1‐nYFP and SEC23A‐cYFP fusion proteins were transiently expressed in Arabidopsis protoplasts, the YFP signal was detected in the cytoplasm (Figure 1A). We also conducted the (BiFC) experiment in *Nicotiana benthamiana*, and reconstituted YFP fluorescence showed that SEC23A, but not SEC23D (used as a control), interacted with SIB1 (Figure S2D). To confirm the interactions between SIB1 and SEC23A, YFP‐SEC23A and SIB1‐RFP were co‐expressed in Arabidopsis protoplasts. Indeed, SIB1 and SEC23A were found to be co‐localized in the cytoplasm under normal conditions (Figure 1B). In addition, a co‐immunoprecipitation (Co‐IP) assay was performed by transiently expressing the SIB1‐FLAG fusion protein together with GFP (control) or the SEC23A‐GFP fusion in *N. benthamiana* leaves. Extracted proteins were incubated with anti‐GFP agarose beads to precipitate SEC23A‐GFP and its associated proteins, and the co‐precipitated proteins were detected using the anti‐FLAG antibodies. As shown in Figure 1C, SIB1‐FLAG specifically interacted with SEC23A‐GFP, but not with free GFP controls. These results provide direct evidence for a physical interaction between SIB1 and SEC23A *in planta*.

### SIB1 is localized to the ER

**Figure 2:**
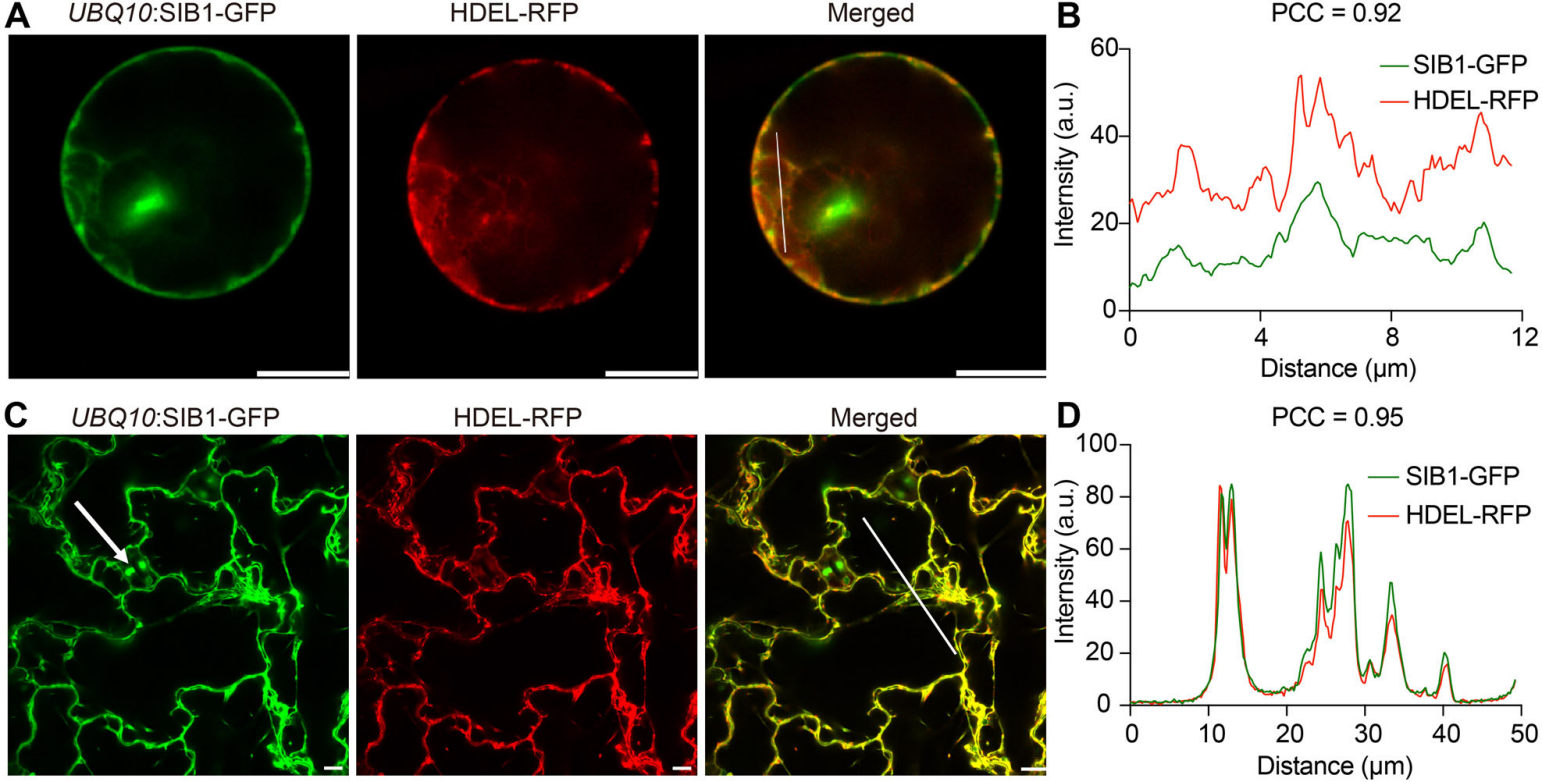
SIB1 is localized to the endoplasmic reticulum (ER) and nucleus **(A)** SIB1‐GFP colocalizes with HDEL‐RFP, which is the ER marker, in Arabidopsis protoplasts. The experiment was repeated three times, with similar results. **(B)** Line scan analysis **(A)** reveals strong spatial colocalization of SIB1‐GFP with the ER marker. **(C)** In pavement cells, SIB1‐GFP colocalizes with HDEL‐RFP–labeled ER. Detached rosette leaves of seedlings from *SIB1‐GFP* transgenic plants in the *HDEL‐RFP* background. The experiment was repeated three times, with similar results. **(D)** Line scan analysis **(C)** reveals strong spatial colocalization of SIB1‐GFP with the ER marker. Scale bar = 10 μm.

SIB1 has previously been reported to be localized in chloroplasts and the nucleus. As SEC23A is known to localize to the ER (Zeng et al., 2015), the interaction of SIB1 with SEC23A indicates that SIB1 could also be localized in the ER. Our BiFC result indicated that SIB1 interacts with SEC23A in the cytoplasm (Figure 1A), raising the possibility that SIB1 might be localized to the ER, which extends through the cytoplasm. To further confirm this result, SIB1 subcellular localization was further analyzed by co‐expressing with the *bona fide* ER marker HDEL‐RFP. Indeed, co‐expression of HDEL‐RFP and SIB1‐GFP in Arabidopsis protoplasts revealed that SIB1 colocalizes with HDEL‐RFP, indicating that it is an ER‐localized protein (Figure 2A). We used confocal microscopy to examine the cell interior. The results demonstrate that the signals co‐localize, forming a network consistent with the endoplasmic reticulum (ER) (Figure S4B). Furthermore, transgenic Arabidopsis plants expressing *UBQ10*:*SIB1‐GFP* were generated and crossed with *HDEL‐RFP* transgenic plants. The localization of the ER marker showed a strong correlation with the SIB1‐GFP distribution in line scan analysis (Figure 2B, D). Consistent with previous studies, SIB1‐GFP showed nucleus localization, whereas HDEL‐RFP showed the typical reticulated pattern of the ER (Figure 2C). In addition to nucleus localization, SIB1‐GFP colocalized with HDEL‐RFP in the transgenic plants, further confirming its ER localization.

### Loss‐of‐function mutation of *SEC23A* compromises resistance to the bacterial pathogen *P. syringae* (*Pst*)

Albeit the role of SIB1 as a positive regulator of plant immunity is known (Xie et al., 2010; Lai et al., 2011), the physiological function of SEC23A in plant stress and immunity remains elusive. The interaction and colocalization between SIB1 and SEC23A resistance raise the possibility that SEC23A may also participate in disease resistance. The Arabidopsis mutant salk_152532c, which contains a T‐DNA insertion in the second exon of *SEC23A*, was obtained from the Arabidopsis Biological Resource Center, and the allele was named *sec23a‐2*. Based on RNA‐seq results (Figure S3A), *sec23a‐2* could only generate a truncated transcript before the insertion site, indicating that it is a knockout mutant. Another T‐DNA insertion line (salk_021996) was obtained from the Nottingham Arabidopsis Stock Centre. This mutant carries the T‐DNA insertion in the promoter region and has been previously reported as a knockdown mutant and was named *sec23a‐1* (Aboulela et al., 2018; Figure S3A). For the *SIB1* locus, two transposon‐tagged lines, *sib1‐3* (SM_3.30601) and *sib1‐4* (SM_3.30596), which were previously reported as knockout lines (Lai et al., 2011), were used in this study for phenotypic analysis. Based on the RNA‐seq results (Figure S3B), the transcripts produced from the *sib1‐4* allele lack the sequence after the insertion site, further indicating that it is a knockout allele.

**Figure 3:**
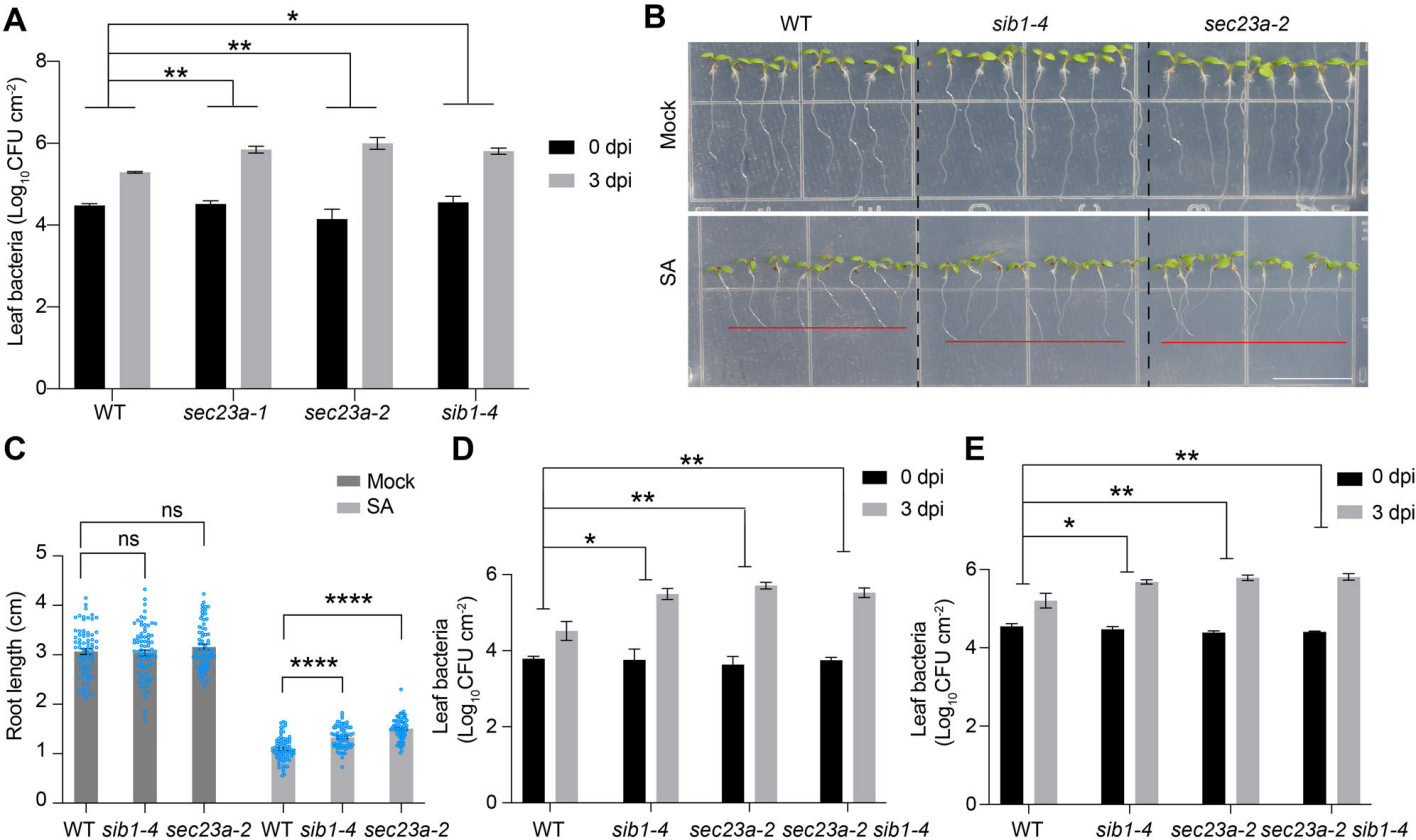
***SIB1 and SEC23A*** **are involved in plant immunity** **(A, D)** Bacterial growth of *Pst* DC3000 on plants of the different genotypes measured at 0 and 3 d post‐inoculation (dpi). The results represent the means ± *SEM* (*n* = 3 biological replicates). Statistical significance: *P < 0.05 and **P < 0.01 (two‐way ANOVA). **(B)** Phenotypic analysis of seedlings of WT, *sib1‐4*, and *sec23a‐2* mutants grown on 1/2MS and 1/2MS plates supplemented with 100 μM SA (sodium salicylate) for 6 d. **(C)** Seedling root length was quantified, and data represent the means ± *SEM* (*n* ≥ 70 seedlings from three biological replicates). ****P < 0.0001 (one‐way ANOVA, followed by Holm–Šídák's multiple comparisons test). ns: not significant. Scale bar = 1 cm. **(E)** Bacterial growth of *Pst* DC3000 *avRpm1* on plants of the different genotypes measured at 0 and 3 dpi. The results represent the means ± *SEM* (*n* = 3 biological replicates). Statistical significance: *P < 0.05 and **P < 0.01 (two‐way ANOVA).

There is no obvious morphological phenotypic difference between these *sec23a* or *sib1* mutants and wild‐type plants under the normal growth conditions. The disease resistance phenotypes of the *sib1* and *sec23a* mutants were further compared with wild‐type plants by infecting them with the virulent strain of the bacterial pathogen *Pst* DC3000 strain. The result showed that *sec23a‐1, sec23a‐2* and *sib1‐4* mutant plants had significantly more bacterial growth than WT, indicating that *SEC23A*, like *SIB1*, also plays a positive role in plant immunity (Figure 3A).

The phytohormone salicylic acid (SA) is accumulated following pathogen infection to induce the plant defense response and plays a crucial role in resistance against a wide range of plant pathogens, including *P. syringae* (Medina‐Puche et al., 2020). To determine whether *sib1 and sec23a* mutations affect the response to SA, seeds of WT, *sib1‐4*, and *sec23a*‐2 were germinated on 1/2MS medium with and without 100 μM SA, which is known to inhibit seedling growth while inducing the defense response. Under normal growth conditions, the 6 d‐old seedlings of *sib1‐4* and *sec23a‐2* mutants showed no significant difference in growth compared to the wild type. However, in the presence of SA, the root length was significantly longer in *sib1‐4* and *sec23a‐2* compared with the wild type, indicating that the *sib1‐4* and *sec23a‐2* mutations reduce sensitivity to SA (Figure 3B, C). To elucidate the genetic relationship between *SIB1* and *SEC23 A*, we generated *sec23a‐2 sib1‐4* double‐mutant lines by crossing the single mutants. No morphological difference was observed among *sec23a‐2*, *sib1‐4*, and *sec23a‐2 sib1‐4* compared with the WT plants under normal growth conditions. Following infection with *Pst* DC3000 and *Pst* DC3000 *avrRpm1*, the s*ec23a‐2 sib1‐4* double mutant showed significantly more growth of both pathogens than WT plants at 3 d post‐inoculation (dpi). *sec23a‐2* and *sec23a‐2 sib1‐4* displayed no significant difference, and both were slightly more susceptible than *sib1‐4* (Figure 3D, E).

### *SIB1 and SEC23A* are involved in the ER stress response

**Figure 4:**
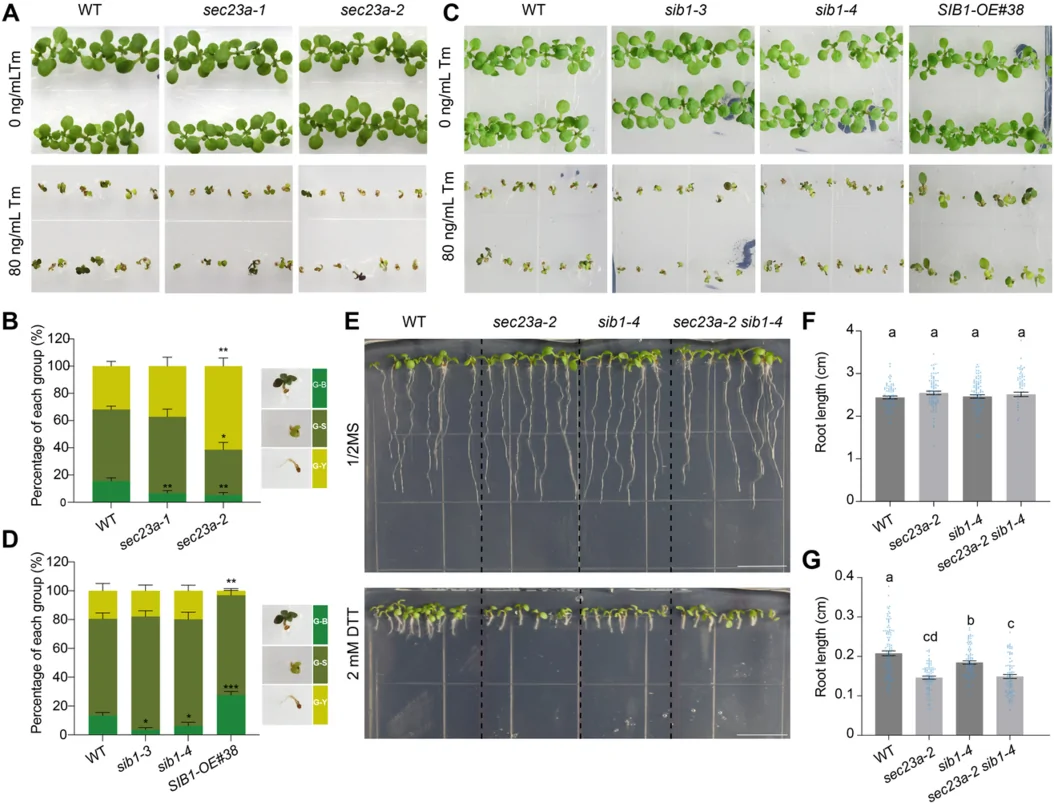
**The mutations in** ***SEC23A*** **and** ***SIB1*** **alter the sensitivity to endoplasmic reticulum (ER) stress** **(A**,** C)** Representative images show 10 d‐old Arabidopsis seedlings grown on either 1/2MS medium or Tm‐supplemented medium. Tm: tunicamycin. **(B**,** D)** Quantitative analysis revealed the distribution of phenotypic classes: green‐big (G‐B), green‐small (G‐S), and yellow‐small (Y‐S) plants (total seedlings ≥ 98 from three independent experiments) treated as indicated in **(A**,** C)**. (one‐way ANOVA, followed by Holm–Šídák's multiple comparisons test) *P < 0.05, **P < 0.01, ***P < 0.001. Unmarked values indicate not significant (ns). **(E)** Representative images show 7 d‐old seedlings grown on 1/2MS medium supplemented with 0 or 2 mM DTT (dithiothreitol). Scale bar = 1 cm. **(F)** Root length of seedlings grown on 1/2MS medium was measured and presented as means ± *SEM* (*n* ≥ 70 seedlings from three biological replicates). The same letter indicates no significant difference in each group (one‐way ANOVA, followed by Holm–Šídák's multiple comparisons test). **(G)** Root length of seedlings grown on 1/2MS medium with 2 mM DTT and presented as means ± *SEM* (*n* ≥ 70 seedlings from three biological replicates). Different letters represent statistically significant differences for each group (*P* < 0.001, one‐way ANOVA, followed by Holm–Šídák's multiple comparisons).

SA has been suggested to induce ER stress signaling and UPR (Nagashima et al., 2014; Lai et al., 2018). We found that tunicamycin (Tm) treatment induced expression of *ProSIB1*: *GUS* in the transgenic lines, as demonstrated by GUS activity assays (Figure S4C). Together with the finding of the localization of SIB1 and SEC23A in the ER, it raises a possibility that they could be involved in the ER stress response. The mutants (*sec23a*, *sib1*) and *SIB1*‐overexpressing transgenic plants (*SIB1‐OE#38*, *SIB1‐OE#9*, Figure S3C, 3D), and WT seedlings were subjected to ER stress by treatment with tunicamycin (Tm), a specific inducer of ER stress. Dithiothreitol (DTT), another chemical inducer of ER stress, was also used in the assay. The *sec23a* and *sib1* mutants grew as well as the wild‐type (WT) control under the normal growth conditions, but they showed enhanced sensitivity to the induced ER stress. The *sec23a‐2* mutation had a stronger effect on ER stress tolerance than the *sec23a‐1* knockdown mutation (Figure 4A, B). The *SIB1‐OE* plants grew as well as the wild‐type (WT) control under the normal growth conditions but showed enhanced tolerance to ER stress (Figures 4C, D, S4D, E). The wild type (WT), *sec23a‐2*, and *sib1‐4* plants showed phenotypes under DTT treatment similar to those observed with Tm treatment. Under normal growth conditions, 7 d‐old seedlings of *sec23a‐2*, *sib1‐4*, and *sec23a‐2 sib1‐4* mutants showed no significant difference in growth compared to the wild type. However, in the presence of DTT, the root length was significantly shorter in all three mutants than that of the wild type (Figure 4E, F, G). There was no significant difference in the root growth inhibition between *sec23a‐2* and *sec23a‐2 sib1‐4,* and both displayed more severe growth inhibition than *sib1‐4*. At the same time, we treated 7 d‐old seedlings with tunicamycin and obtained results similar to those observed with DTT treatment (Figure S4G, H). The results showed that the phenotypic outcomes observed at 7 d (Figure S4G, H), although milder, showed a similar trend to those seen at 10 d (Figure 4A–D). We then tested ER stress sensitivity in *sec23a‐2*, *sib1‐4*, and *sec23a‐2 sib1‐4* adult plants by injecting leaves with tunicamycin. As shown in Figure S4A, leaves of *sec23a‐2*, *sib1‐4*, and *sec23a‐2 sib1‐4* senesced more rapidly than WT. The double mutant *sec23a‐2 sib1‐4* showed a senescence rate similar to that of *sec23a‐2* but faster than that of *sib1‐4*.

The above results indicated that both SIB1 and SEC23A are involved in the defense response and the ER stress response, and SEC23A might act downstream.

### SIB1 and SEC23A accumulate in chloroplasts under stress

**Figure 5:**
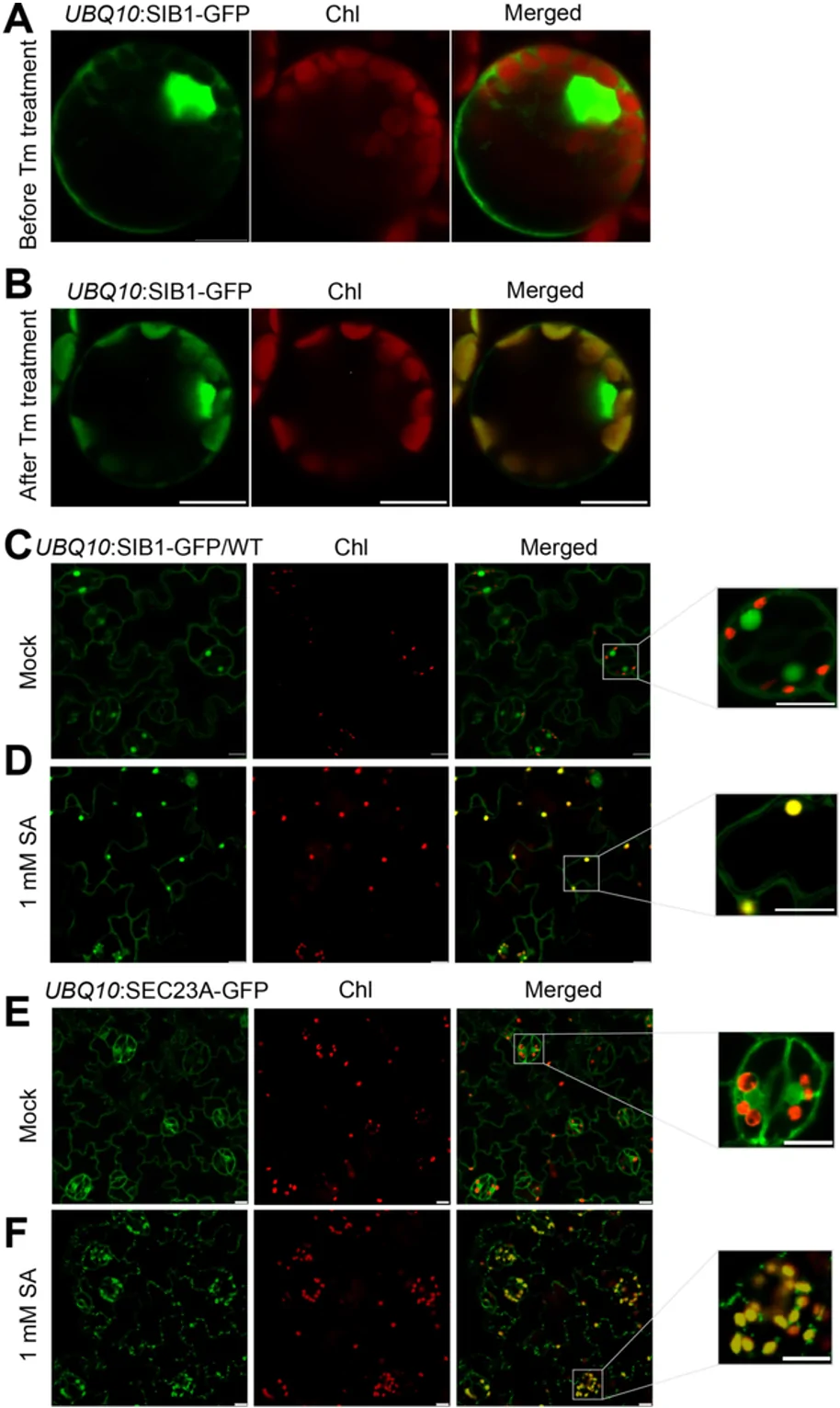
SIB1 and SEC23A accumulate in chloroplasts under stress **(A)** Before Tm treatment, SIB1‐GFP was detected in the nucleus and the ER. Tm: tunicamycin. **(B)** After Tm treatment for 6 h, SIB1‐GFP was detected in the chloroplasts. **(C**, **D)** Subcellular localization of SIB1‐GFP in detached leaves of 6 d‐old seedlings of *SIB1‐GFP* transgenic plants in the WT. Before SA (sodium salicylate) treatment, SIB1‐GFP was detected in the nucleus and the ER. After CHX (50 μM, 2 h prior to 1 mM SA treatment) and SA treatment for 21 h, SIB1‐GFP was detected in the chloroplasts in the WT background. CHX: cycloheximide. **(E)** In a leaf from a 6 d‐old *SEC23A‐GFP* seedling before SA treatment, SEC23A‐GFP was detected in the ER but not in chloroplasts. **(F)** After CHX (50 μM, 2 h prior to 1 mM SA treatment) and SA treatment for 21 h, SEC23A‐GFP was detected in the chloroplasts. The experiments were repeated three times, with similar results. Chl: chloroplasts. Scale bar = 10 μm.

SIB1 is known to interact with SigA in chloroplasts. However, under normal conditions, we found that SIB1 was associated with the ER and the nucleus, but was not detected in chloroplasts (Figure 2). To test if SIB1 might change its subcellular localization under ER stress, *UBQ10:*SIB1‐GFP was expressed in Arabidopsis protoplasts upon ER stress induced by Tm treatment. Consistent with the previous observation under normal conditions, when expressed in Arabidopsis protoplasts, the SIB1‐GFP signal was observed in the nucleus and the cytosol but not in chloroplasts (Figure 5A). Notably, the SIB1‐GFP signal was prominently found in the chloroplasts and the nucleus upon ER stress (Figure 5B).

Stable transgenic lines carrying *UBQ10:SIB1‐GFP* were generated in both WT and *sec23a‐2* backgrounds to test if the SIB1 translocation from the ER to chloroplasts requires SEC23A. Under normal conditions, SIB1‐GFP was observed in the nuclei and the cytosol but not in chloroplasts in both WT and *sec23a‐2* plants. However, the SA treatment triggered the accumulation of SIB1‐GFP in chloroplasts in WT as well as in *sec23a*, while its localization in the cytosol was decreased (Figures 5C, D, S5A). In this experiment, the plants were also treated with the translation inhibitor cycloheximide (CHX) prior to 1 mM SA treatment to block new protein synthesis. The result indicates that localization of SIB1‐GFP in chloroplasts under ER stress likely occurs through its re‐location from the ER to chloroplasts, instead of through targeting of newly synthesized protein to chloroplasts. This shuttling might not require trafficking through the SEC23A‐involved COPII vesicles, since it still occurred in the *sec23a‐2* mutant. Alternatively, SEC23A‐containing vesicles could be involved in the shuttling, but due to functional redundancy, the *sec23a* mutation did not block the relocation of SIB1 from the ER to chloroplasts under SA treatment.

Under normal conditions, SEC23A‐GFP was found to be localized on the ER (Figure 5E). When YFP‐SEC23A and SIB1‐RFP were co‐expressed in Arabidopsis protoplasts, they were found to be co‐localized in the cytoplasm, whereas SIB1 was also found in the nucleus (Figure 1B). The finding of relocalization of SIB1 to chloroplasts under ER stress and the interaction between SEC23A and SIB1 raise the possibility that SEC23A could also localize to chloroplasts under stress conditions. Multiple subcellular prediction algorithms do predict that SEC23A (At4g01810) contains a signal peptide that might target it to chloroplasts (Figure S5B). To test this experimentally, the *SEC23A‐GFP* transgenic line was treated with SA; the SEC23A‐GFP protein was indeed localized to chloroplasts upon the treatment (Figure 5E, F). Following pathogen infection, both SIB1 and SEC23A proteins accumulate in chloroplasts (Figure S6A, B).

### Transcriptome profiling analysis reveals the role of *SIB1* and *SEC23A* in the expression of defense transcriptomes

**Figure 6:**
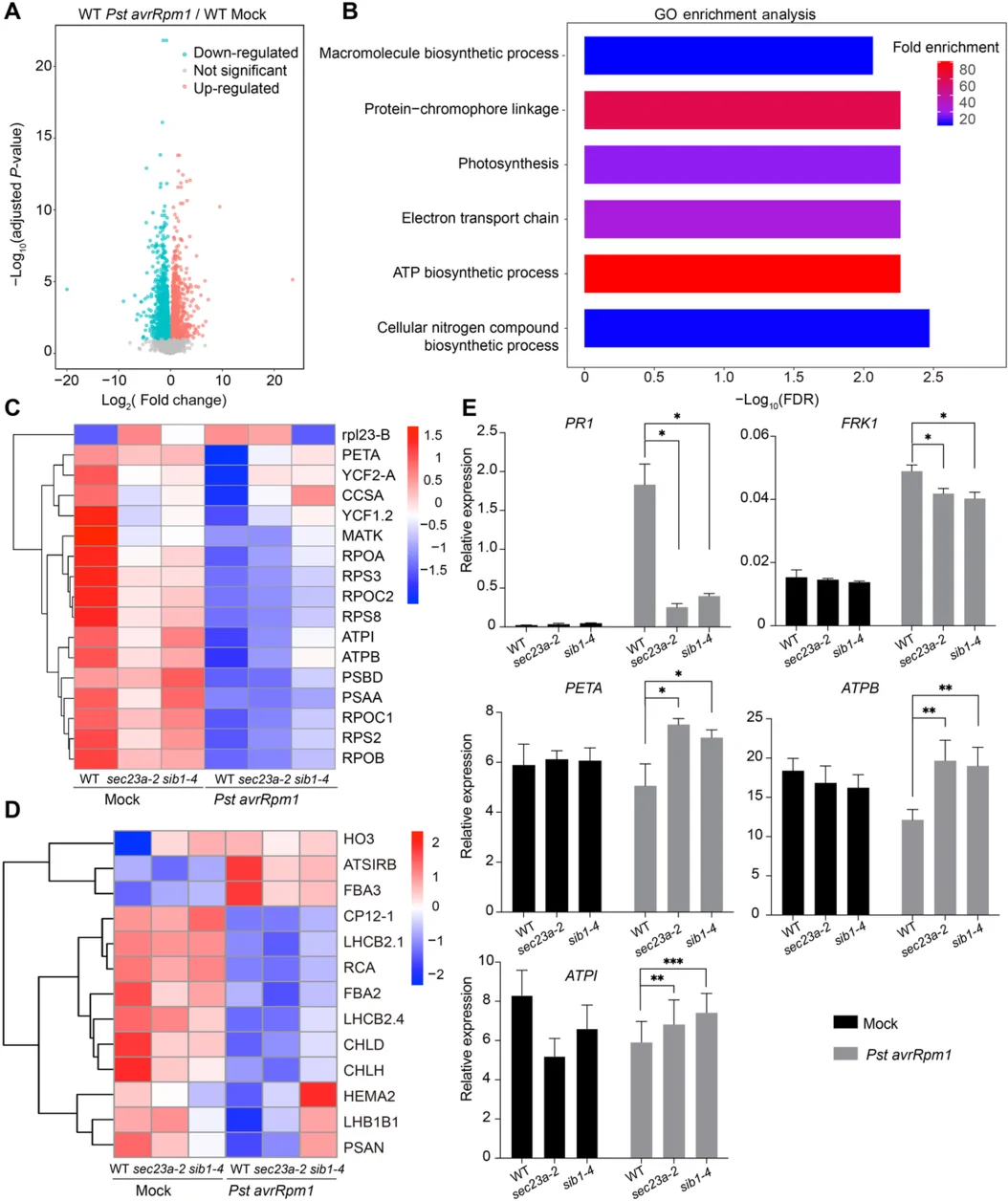
**Transcriptome analysis of expression profiles in WT,** ***sec23a*,** **and** ***sib1*** **plants before and after pathogen infection** **(A)** Transcriptome analysis of pathogen‐responsive genes. Col‐0 leaves were sprayed with *Pst avrRpm1* or Mock (MgCl_2_). Total RNA was extracted 3 h later and subjected to RNA sequencing. Shown in the *x*‐axis are Log_2_‐fold changes of transcripts in *Pst avrRpm1*‐treated plants compared with mock. A significant change (adjusted *P*‐value < 0.05) appears in blue (downregulated genes) or red (upregulated genes), and gray means no DEGs. Pathogen‐responsive genes: adjusted *P*‐value < 0.05. **(B)** Gene ontology (GO) terms for chloroplast‐encoded genes among the pathogen‐responsive genes. **(C)** Heatmap showing the changes in transcript levels of chloroplast‐encoded genes in WT, *sec23a‐2*, and *sib1‐4* before and after pathogen infection. **(D)** Heatmap showing the changes in transcript levels of photosynthesis‐associated nuclear genes (PhANGs) of WT, *sec23a‐2*, and *sib1‐4* before and after pathogen infection (showing the top upregulated and downregulated genes). The results shown above were averaged from three biological replicates. **(E)** RT‐qPCR analysis of expression of *PR1*, *FRK1*, *PETA*, *ATPB*, and *ATPI* in *sib1‐4* and *sec23a‐2* plants. Statistical analysis was performed using a paired *t*‐test. *n* = 6 biological replicates. *P < 0.05, **P < 0.01, and ***P < 0.001.

Mutation of *SIB1* has been reported to affect the expression of chloroplast‐ and nucleus‐localized photosynthesis‐related genes (Lv et al., 2019). However, the previous transcriptome analysis comparing the mutant and WT used poly(A)‐enriched RNA samples, which could not reflect the chloroplast transcriptome profiles. Additionally, the effect of the *SEC23A* mutation on defense gene regulation remains unknown. To further investigate the roles of *SIB1* and *SEC23A* in mediating the expression of both nuclear and chloroplast genes, RNA‐sequencing (RNA‐seq) of WT and the *sib1‐4* and *sec23a‐2* mutants was conducted with or without pathogen infection. The plants were infected with the avirulent *P. syringae* strain DC3000 expressing the avirulent gene *avrRpm1* (*Pst avrRpm1*) and the infected leaves were collected 3 h after pathogen infection for RNA extraction to analyze the role of *SIB1* and *SEC23A* in gene expression during the defense response. After rRNA depletion of the total RNA samples, cDNA libraries were prepared for sequencing using the Illumina's second‐generation sequencing technology and the transcriptome data were analyzed using DESeq 2. A total of 2,753 genes were found to show significant differential expression (DEGs) (Benjamini–Hochberg‐adjusted *P‐*value < 0.05) (Wang et al., 2022) between *Pst avrRpm1*‐inoculated WT and mock‐inoculated WT, with 1,289 upregulated genes and 1,464 downregulated genes (Figure 6A; Table S2). These genes are referred to as pathogen‐responsive genes in this study. After pathogen infection, the expression patterns of the pathogen‐responsive genes in the *sec23a‐2* and *sib1‐4* mutants largely mirrored those in the wild type (WT) (Figure S7), with most of the upregulated and downregulated genes maintaining their trends. However, a small subset of genes showed divergent expression. In *sec23a‐2* and *sib1‐4*, a subset of pathogen‐responsive upregulated genes showed downregulation, while some pathogen‐suppressed genes showed upregulation. This transcriptional dysregulation likely contributes to the enhanced pathogen susceptibility observed in these mutants.

Among the pathogen‐responsive genes, chloroplast‐encoded genes were predominantly downregulated following pathogen infection in WT (Figure 6C). As expected for chloroplast genes, Gene ontology (GO) term analysis showed that these DEGs were enriched in the photosynthesis function (Figure 6B). Most of these genes were also downregulated in the *sec23a* and *sib1* mutants following pathogen infection, but to a much lesser extent. These results indicated that *SEC23A* and *SIB1* might work to suppress the expression of photosynthesis‐associated plastid genes (PhAPGs) in response to pathogen infection. However, under normal conditions, these genes were mostly expressed at lower levels in the *sec23a* and *sib1* mutants compared to the wild type. As SIB1 and SEC23A were not detected in chloroplasts under normal conditions, it is possible that other factors might contribute to the reduced expression of these chloroplast genes when SEC23A or SIB1 are not functional (Figure 5C, E).

Among the pathogen*‐*responsive genes, the photosynthesis‐associated nuclear genes (PhANGs) were also mostly downregulated after pathogen infection in the wild type, indicating that pathogen infection led to an overall reduction of photosynthetic genes. Similarly, the *sec23a* and *sib1* mutants also generally displayed reduced expression of these PhANGs in response to the infection compared to the mock condition. However, the reduction of their expression upon pathogen infection was less prominent compared to WT. In addition, under the mock conditions, these genes also showed a reduced expression in the *sec23a* and *sib1* mutants compared to the WT (Figure 6D). These results indicated that reduced expression of photosynthetic genes in the response to pathogen infection might be an active defense mechanism and that *SIB1* and *SEC23A* play a role in this mechanism. The result is consistent with the previous reports that pathogen infection leads to suppression of photosynthesis (Bonfig et al., 2006; Berger et al., 2007; Lu and Yao, 2018). To further validate the transcriptome profiling results, the pathogen infection experiment was repeated in Arabidopsis, and the total RNA samples extracted from the plants were analyzed by RT‐qPCR. *PR1* was strongly induced by pathogen infection; however, the *sib1‐4* and *sec23a‐2* mutants showed weaker *PR1* induction than WT. *FRK1* (*FLG22‐INDUCED RECEPTOR‐LIKE KINASE 1*), another defense‐related marker gene, was also rapidly induced but showed weaker expression in s*ib1‐4* and *sec23a‐2* mutants than in the WT. The expression patterns of the *PETA*, *ATPB*, and *ATPI* genes, involved in photosynthesis, were found to be consistent with the RNA‐Seq findings (Figure 6E). After pathogen infection, their transcript levels in mutants were higher than that in the wild type. Based on the RNA‐seq results, among the pathogen‐responsive genes, ER stress‐related genes such as *PDI5*, *PDI6*, and *CRT2* were upregulated in WT following pathogen infection. These genes also showed upregulation in the *sec23a‐2* and *sib1‐4* mutants, though to a much lesser extent (Figure S4F). These findings support the involvement of SIB1 and SEC23A in ER stress responses triggered by pathogen infection.

## DISCUSSION

Inter‐organelle communication has increasingly been recognized as a crucial aspect of cellular stress responses and homeostasis (Liu and Howell, 2010; Liu and Li, 2019; Depaepe et al., 2021). Various unfavorable environmental conditions, such as pathogen infections, can induce stress symptoms in different organelles, including ER stress characterized by the unfolded protein response and the production of reactive oxygen species in chloroplasts. However, the mechanisms by which these organelles communicate to balance stress responses and growth remain largely unknown.

SIB1 has previously been reported to be localized in chloroplasts and the nucleus, where it regulates the expression of plastid and nuclear genes in response to pathogen infection (Xie et al., 2010; Lai et al., 2011; Lv et al., 2019). Our study identified SEC23A (At4g01810), an essential component of the COPII complex involved in membrane trafficking between the ER and the Golgi apparatus, as an interacting partner of SIB1. This discovery led us to find that SIB1 also co‐localizes with SEC23A in the ER under normal conditions. The previous failure to detect SIB1 in the ER is likely due to its low abundance under normal conditions. Under the ER stress including treatment with SA, we found that both SIB1 and SEC23A re‐localized to chloroplasts and were barely detectable in the ER. Our findings also indicate that SEC23A, like SIB1, plays a role in plant immune responses, while both SIB1 and SEC23A are involved in the ER stress response. This study suggests that SIB1 is associated with SEC23A on the surface of the ER under normal conditions. Once stress is perceived, SIB1 is released into the chloroplasts, where it inhibits the expression of chloroplast‐encoded photosynthesis‐related genes.

**Figure 7:**
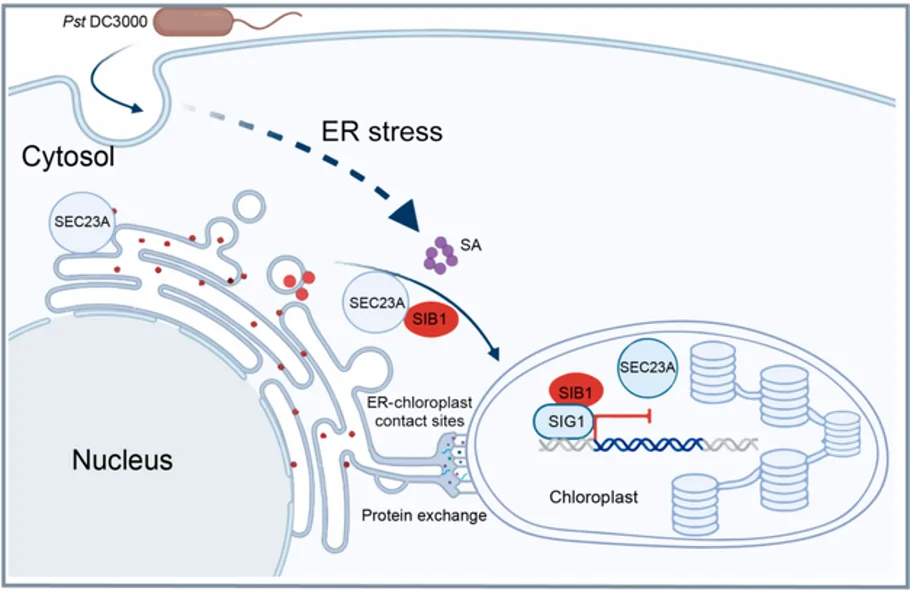
Hypothetical working model In plant cells, SIB1 and SEC23A are associated with the endoplasmic reticulum. SIB1 could interact with SEC23A. On encountering stress, SIB1 is released into the chloroplasts in a process mediated by SEC23A.

The mechanism underlying the relocalization of SIB1 and SEC23A from the ER to chloroplasts remains to be defined. One possibility is that ER stress and pathogen infection trigger the formation of SEC23A‐containing vesicles that transport SIB1 from the ER to chloroplasts, analogous to the ER–Golgi vesicle trafficking (Figure 7); however, ER–chloroplast vesicle trafficking has not yet been reported. It is known that the ER forms contact sites with various organelles, including chloroplasts (Liu and Li, 2019; Bian et al., 2023), and therefore, it is possible that proteins such as SIB1 and SEC23A might be transported between the ER and chloroplasts through these contact sites.

Chloroplasts and photosynthesis play crucial roles in plant immunity. It has been reported that during pathogen infection, chloroplasts may downregulate photosynthesis to prioritize the production of defense‐related metabolites, such as salicylic acid and other phytoalexins (Serrano et al., 2016). The perception of microbe‐associated molecular patterns (MAMPs) by host MAMP receptors triggers the suppression of nuclear‐encoded chloroplast‐targeted genes (NECGs). Conversely, some virulent effectors of *P. syringae* target chloroplasts to prevent reactive oxygen bursts and activation of the immune response (de Torres Zabala et al., 2015). We also found that pathogen infection led to a reduction in the expression of most chloroplast genes and some nuclear genes associated with photosynthesis. However, mutations in *sib1* and *sec23a* compromise the reduction in the expression of these genes. This finding suggests that SIB1 and SEC23A normally respond to pathogen infection by targeting chloroplasts to alter the expression of photosynthesis‐related genes. The resulting changes in the normal photosynthesis process could lead to the production of ROS and enhanced expression of defense genes. A comprehensive understanding of this ER–chloroplast interaction could provide deeper insights into the integrated networks of inter‐organelle communication in plant immunity and other stress responses.

## MATERIALS AND METHODS

### Plant materials and growth conditions

The wild type utilized in this study was the Columbia (Col‐0) ecotype of *Arabidopsis thaliana*. The single mutants *sec23a‐1* (salk_021996), *sec23a‐2* (salk_152532c), *sib1‐3* (SM_3.30601), and *sib1‐4* (SM_3.30596) were acquired from the Arabidopsis Biological Resource Center. Arabidopsis seeds were surface‐sterilized (75% ethanol + 0.05% Triton X‐100, 10 min), washed with sterile water, and sown on 1/2MS medium (1% sucrose, 0.8% agar). After stratification (4°C, 3 d in darkness), seedlings were grown in a controlled chamber (16 h light/8 h dark, 22°C). The plants for the pathogen inoculation assay were grown directly in soil under a light/dark cycle of 15 h of light, followed by 9 h of darkness in a growth room at 22°C with a humidity level of 50%. *Nicotiana benthamiana* plants were cultivated directly in soil at a temperature of 24°C under long‐day conditions. For transgenic overexpression plants, the binary vectors pCAMBIA1300, pCAMBIA1305, and pBI121 were engineered to harbor the coding sequences of *SIB1* and *SEC23 A*, both under control of the *UBQ10* promoter. *Agrobacterium tumefaciens* GV3101 was transformed with the constructs, and transgenic plants were generated via floral dip. Double‐transgenic lines were created through genetic crosses. To generate *ProSIB1*:*GUS* transgenic plants, the promoter of *SIB1* were inserted into the pGWB533 vector. Refer to Table S3 in the Supporting Information for primer sequences.

### Yeast two‐hybrid assay

The yeast two‐hybrid screening assay was conducted by Hybrigenics Services, S.A.S. After amplification by PCR, the DNA segment corresponding to *A. thaliana* SIB1 residues 1–100 was ligated into the pB29 plasmid (derived from the pBTM116 vector) (Vojtek and Hollenberg, 1995; Beranger et al., 1997), yielding an N‐terminal LexA chimeric protein (SIB1‐LexA). The construct was checked by sequencing and used as a bait to screen a random‐primed Arabidopsis *Pseudomonas*‐infected cDNA library constructed into pP6. The prey fragments of positive clones were amplified by PCR and sequenced at their 5’ and 3’ junctions. The resulting sequences were used to identify the corresponding interacting proteins in the GenBank database (NCBI) using a fully automated procedure. For yeast two‐hybrid targeted assays, the pGBKT7 vector was used to clone the coding sequences of *SIB1*. The coding sequences of *SEC23A* were cloned into the pGADT7 vector. The vectors that were acquired were co‐transformed into the yeast strain AH109. The yeast transformants were individually plated onto SD medium: non‐selective medium lacking Trp and Leu (2D), selective medium lacking Trp, Leu, and His (3D), and selective medium lacking Trp, Leu, His, and Ade (4D), and incubated at 28°C for about 2–3 d.

### Pathogen infection and disease resistance assays

Three to 4‐week‐old short‐day grown well plants were spray‐inoculated with either virulent *P. syringae* (*Pst*) DC3000 or avirulent *Pst* DC3000 *avrRpm1* (OD_600_ = 0.05 in 10 mM MgCl_2_ with 0.02% Silwet L‐77). The plants were subsequently enclosed in a clear plastic dome to maintain humidity for a duration of 24 h. Bacterial proliferation was observed 3 d post‐inoculation.

### Co‐immunoprecipitation (Co‐IP) assay

Co‐IP assays were used to study the intracellular interaction between SEC23A and SIB1 in plant cells. Leaves of *N. benthamiana* were transiently co‐expressed with *UBQ10:*SIB1‐3×Flag, *35S:*SEC23A‐GFP, and *35S:*GFP. Following a 2 d incubation period, the leaves that had been collected were subjected to grinding in liquid nitrogen to ensure proper fragmentation. Subsequently, the ground material was resuspended in an extraction buffer formula. The buffer contained 10 mM Tris‐HCl (pH 7.5), 2 mM EDTA, 300 mM NaCl, and 10% glycerol. The mixture was vortexed vigorously at 4°C for 15 min to ensure efficient extraction. Total leaf proteins (200 mg) were immunoprecipitated with anti‐GFP‐conjugated agarose beads at 4°C for 2 h. Following three PBS washes, the bound proteins were eluted by heating in SDS buffer. Electrophoretic separation was performed using SDS‐PAGE, followed by immunodetection using anti‐GFP (Abmart, M20004M) and anti‐Flag (Sigma, M2, monoclonal) antibodies.

### Bimolecular fluorescence complementation assay

In the bimolecular fluorescence complementation (BiFC) assay system, the N‐terminal portion of the yellow fluorescent protein was fused with both SIB1 and SEC23A using the pSAT6‐nEYFP and pSAT6‐cEYFP vectors, respectively. These constructs were transiently co‐expressed in the mesophyll protoplasts of Arabidopsis. Isolation of protoplasts was performed following a method outlined by Yoo et al (Yoo et al., 2007). Confocal microscopy images were acquired using a Leica Stellaris system. The vectors used for BiFC in *N. benthamiana* are pGTQL1211YN and pGTQL1221YC.

### Subcellular localization analysis

Protoplasts from Arabidopsis mesophyll tissues were isolated and subjected to transfection using the previously described method (Yoo et al., 2007). In this assay, PBI221 vectors carrying SIB1 and HDEL were C‐terminally fused with RFP or GFP and SEC23A was N‐terminally fused with YFP under control of the *UBQ10* promoter. Confocal imaging was carried out with a laser‐scanning microscope system (Leica Stellaris). RFP fluorescence and GFP (YFP) and autofluorescence were stimulated using lasers of 561 nm and 488 nm, respectively, and captured using spectral detectors configured with suitable detection ranges. Image analysis was conducted using Fiji software. The above‐described approach is equally suitable for transgenic plant lines.

### Histochemical GUS activity analysis

Seven‐day‐old *ProSIB1:GUS* transgenic seedlings were treated with or without 5 μg/mL Tm for 24 h before β‐glucuronidase (GUS) histochemical staining. Histochemical staining was performed using a GUS Stain Kit (Solarbio, Cat. No. G3060), followed by destaining and observation.

### RNA extraction and quantitative reverse transcription‐PCR (RT‐qPCR)

Arabidopsis tissue samples were subjected to total RNA extraction using the RNeasy Plant Mini Kit (QIAGEN), incorporating an on‐column DNase I step for genomic DNA removal. Following quality assessment, total RNA (1 μg) was subjected to treatment with gDNA Eraser (Takara) and reverse‐transcribed using PrimeScript RT reagent. Quantitative PCR reactions (20 μL volume) were carried out using the cDNA template, SYBR Green master mix (Vazyme), and gene‐specific primers on an ABI 7500 Real‐Time PCR System with standard cycling parameters. The relative expression levels were normalized to *ACTIN2*.

### RNA‐seq library construction and sequencing

The RNA‐Seq library construction and sequencing services were provided by Novogene Co., Ltd. (Beijing, China), with three biological replicates per sample. A total RNA preparation was cleaned of ribosomal RNA, followed by ethanol precipitation. After fragmentation, random hexamer primers were applied for the synthesis of the first‐strand cDNA. In the process of synthesizing the second‐strand cDNA synthesis, dUTPs were substituted with dTTPs in the reaction mix. The directional library was prepared following the completion of end repair of the DNA fragment, A‐tailing of the DNA fragment, adapter ligation of adapters, size selection, USER enzyme digestion, amplification of the ligated products, and purification steps. The library was subjected to assessment using Qubit, followed by quantification of real‐time PCR and use of a bioanalyzer for detecting fragment size distribution. Following quality assessment and quantification, the libraries were combined and loaded on Illumina NovaSeq6000 platforms for a PE150 read length, and over 12 G of raw data per sample were generated.

### RNA‐seq data analysis

Following adapter removal, RNA‐seq reads were subjected to genomic alignment with STAR (v2.5.3a) against the Arabidopsis reference genome (Ensembl release 41; Aken et al., 2016). Alignment parameters followed the original publication (Dobin et al., 2013), utilizing default configurations. Gene expression levels were quantified from STAR‐aligned reads from BAM files using RSEM v1.2.30 (Li and Dewey, 2011) with default settings. For differential analysis, we applied DESeq2 to compare transcriptome profiles between mutant and wild‐type samples, with genes meeting the criterion of adjusted *P*‐value < 0.05 being identified as differentially expressed genes (DEGs) (Lönnstedt and Speed, 2002), with differentially expressed genes (DEGs) defined by an adjusted *P‐*value < 0.05. DEGs identified through RNA‐seq were subjected to functional enrichment analysis. GO terms and biological pathways were assessed using ShinyGO 0.82 (Ge et al., 2020). Only terms with an FDR < 0.05 were considered statistically significant.

### Generative AI usage statement

During the preparation of this work, the authors utilized ChatGPT for the purpose of language editing. After using ChatGPT, the authors carefully reviewed and revised the content, where necessary, and take full responsibility for the final version of the publication.

## CONFLICTS OF INTEREST

The authors declare no conflicts of interest.

## AUTHOR CONTRIBUTIONS

Y.X., J.P., and L.J. developed the research plan and designed the experiments. J.P. performed the experiments with assistance from J.Z., H. Zhang., W.L., and Y.Z. H. Zhong performed the bioinformatic analysis. J.P., H. Zhong., and Y.X. analyzed the data. All authors contributed to manuscript writing and approved the manuscript.

## Supporting information

Additional Supporting Information may be found online in the supporting information tab for this article: http://onlinelibrary.wiley.com/doi/10.1111/jipb.70251/suppinfo

**Figure S1.** The N‐terminus of SIB1, comprising the first 100 amino acids, lacks transcription‐activating ability in the yeast two‐hybrid system
**Figure S2.** SIB1 interacts with SEC23 A in Y2H and BiFC assays
**Figure S3.** Mutant identification
**Figure S4.** *SIB1* and *SEC23 A* may be involved in endoplasmic reticulum (ER) stress
**Figure S5.** Stress conditions induce the accumulation of SIB1 and SEC23 A proteins in chloroplasts
**Figure S6.** SIB1 and SEC23 A accumulate in chloroplasts following pathogen infection
**Figure S7.** Overall expressions of pathogen‐responsive genes were affected by the mutations compared to the wild type

**Table S1.** Results of the yeast two‐hybrid screening
**Table S2.** RNA‐Seq results of pathogen‐responsive genes related to Figure 6
**Table S3.** List of primers used in this study

## Data Availability

The raw RNA sequencing data are available in the NCBI Gene Expression Omnibus (GEO) under accession number GSE300417. Sequence data for the genes described in this article can be found in The Arabidopsis Information Resource (TAIR) under the following numbers: SIB1 (AT3G56710), SEC23A (AT4G01810), SEC23D (AT2G27460), PR1 (AT2G14610), FRK1 (AT2G19190), PETA (ATCG00540), ATPB (ATCG00480), ATPI (ATCG00150), CRT2 (AT1G09210), PDI5 (AT1G21750), and PDI6 (AT1G77510).
