# Supplementary material for: SIB1‐SEC23A undergo ER to chloroplast relocalization to mediate immunity in Arabidopsis thaliana
Source: J Integr Plant Biol. 2026 Apr 12;68(9):3458–72. doi: 10.1111/jipb.70251 (PMC13545851; doi:10.1111/jipb.70251)
Supplement: Supplementary file 1 [file JIPB-68-3458-s001.docx]

**SIB1-****SEC23A undergo ER to chloroplast** **relocalization to mediate immunity in *Arabidopsis thaliana***

**SUPPORTING INFORMATION**

**Figure S1. The N-terminus of SIB1, comprising the first 100 amino acids, lacks transcription-activating ability in yeast two-hybrid system**

(A) Schematic representation of the various regions of the SIB1 protein. aa: amino acid. (B) Yeast cell growth containing different constructs after 2 days on SD/−Leu/−Trp agar plates and 4 days on SD/−Leu/−Trp/−His/−Ade agar plates. The yeast cultures underwent serial dilution as specified, with empty vectors used as the negative controls.

**Figure S2. SIB1 interacts with SEC23A in Y2H and BiFC assays**

(A) Schematic representation of SEC23A truncated proteins. SID: Selected Interaction Domain. aa: amino acid. (B) Growth on SD/−Leu/−Trp agar plates for 2 days; Results on SD/−Leu/−Trp/−His+1mM 3AT agar plates after 4 days. Empty vectors were used as negative controls. (C) SEC23A-SIB1 complex structure predicted with AlphaFold3. SEC23A (1-880 aa) was colored in blue and pink. SIB1 (1-151 aa) was colored in grey and green. Green: VQ motif of SIB1, Pink: Trunk domain of SEC23A. aa: amino acid. (D) Bimolecular fluorescence complement (BiFC) assays were performed by co-infiltrating *N. benthamiana* leaves with *A. tumefaciens* strains expressing the target constructs. YFP reconstitution was imaged at 48 h post-infiltration.SEC23D served as the negative control. YC, C-terminus of YFP; YN, N-terminus of YFP. Scale bar = 10 μm.

**Figure S3. Mutants identification**

(A) Illustration of *AtSEC23A* with T-DNA insertion sites in genome. Black boxes represent exons. Integrated genomics image of transcripts of salk_152532 based on RNA-Seq results. (B) Structures and mutants for *SIB1* and integrated genomics image of transcripts of *sib1-4* based on RNA-Seq results. (C) (D) RT-qPCR analysis of SIB1 mRNA expression levels in wild-type and SIB1 overexpressing transgenic lines (*UBQ10:SIB1-3XFLAG#38*, *UBQ10:SIB1-3XFLAG#9*). n= 4 biological replicates, *****P* < 0.0001(t-test).

**Figure S4.** ***SIB1* and *SEC23A* may be involved in Endoplasmic reticulum (ER) stress**

(A) Leaves from six-week-old plants were infiltrated with tunicamycin (Tm, 1 μg/mL) using a needleless syringe and imaged three days post-treatment. Scale bar = 1cm. (B) In *Arabidopsis* protoplasts, SIB1-GFP exhibits colocalization with the endoplasmic reticulum (ER) marker HDEL-RFP. Bar=10 μm. (C) Seven-day-old *ProSIB1:* GUS transgenic seedlings were treated with or without 5μg/mL Tm for 24h before β-glucuronidase (GUS) histochemical staining. Data are representative of three independent experiments. Scale bar = 0.1 cm. (D) Representative images show 10-day-old *Arabidopsis* seedlings grown on either 1/2MS medium or Tm-supplemented medium. Tm: tunicamycin. (E) Quantitative analysis revealed the distribution of phenotypic classes: green-big (G-B), green-small (G-S), and yellow-small (Y-S) plants (total seedlings ≥ 97 from three independent experiments) treated as indicated in **(D)**. (one-way ANOVA followed by Holm-Šídák’s multiple comparisons test) **P* < 0.05, *****P* < 0.0001. Unmarked values indicate not significant (ns) . (F) Heat map showing the changes in transcript levels of *CRT2*(*AT1G09210*) , *PDI5*(*AT1G21750*), *PDI6*(*AT1G77510*) genes in WT, *sec23a-2* and *sib1-4* before and after pathogen infection. The results are averaged from three biological replicates. (G) Representative images show 7-day-old *Arabidopsis* seedlings grown on either 1/2MS medium or Tm-supplemented medium. Tm: tunicamycin. (H) Quantitative analysis revealed the distribution of phenotypic classes: green-big (G-B), green-small (G-S), and yellow-small (Y-S) plants (total seedlings ≥ 97 from three independent experiments) treated as indicated in **(G)**. (one-way ANOVA followed by Holm-Šídák’s multiple comparisons test) **P* < 0.05, ***P* < 0.01, ****P* < 0.001, *****P* < 0.0001. Unmarked values indicate not significant (ns).

**Figure S5.** **Stress conditions induce the accumulation of SIB1 and SEC23A proteins in chloroplasts**

(A) Subcellular localization of SIB1-GFP in detached leaves of 6-day-old seedlings of SIB1-GFP transgenic plants in the *sec23a-2* backgrounds. Before SA (sodium salicylate) treatment, SIB1-GFP was detected in the nucleus and Endoplasmic Reticulum (ER). After CHX (50 μM, 2 h prior to 1mM SA treatment) and SA treatment for 21 h, SIB1-GFP was detected in the chloroplasts. CHX: cycloheximide, Chl: Chloroplasts, Scale bar = 10 μm. (B) Prediction of subcellular localization of SEC23A using the protein prediction server at https://aramemnon.botanik.uni-koeln.de/.

**Figure S6****.** **SIB1 and SEC23A accumulates in chloroplasts following pathogen infection**

(A) *Pst DC3000 avrpm1* infection alters the subcellular localization of SIB1. SIB1-GFP was transiently expressed in *N. benthamiana* leaves infiltrated with *Pst DC3000 avrpm1* (OD_600_ = 0.05) or mock inoculated for 18 h. (B) *Pst DC3000 avrpm1* infection alters the subcellular localization of SEC23A. SEC23A-GFP was transiently expressed in *N. benthamiana* leaves infiltrated with *Pst DC3000 avrpm1* (OD_600_ = 0.05) or mock inoculated for 18 h. Chl:chloroplasts. Scale bar = 10 μm.

**Figure S7. Overall expression of** **pathogen-responsive genes were affected by the mutations compared to wild type**

(A) Heat map showing changes in the expression of pathogen-responsive genes that were up-regulated in the *sec23a-2* and *sib1-4* mutants. (B) Heat map showing changes in the expression of pathogen-responsive genes that were down-regulated in the *sec23a-2* and *sib1-4* mutants. The results shown above are averaged from three biological replicates.

**Table S1.** **Results of the yeast two-hybrid screening**

|  | **Value** | **Comment** |
| --- | --- | --- |
| Preys tested clones | 81 million | 8-fold the complexity of the library |
| Positive clones | 266 |  |
| Protein identified | 54 |  |

**Table S2.** **RNA-Seq results of** **pathogen-responsive genes related to Figure 6**

| **Gene ID** | **Annotation** | **number of positive clones** | **Global PBS** |
| --- | --- | --- | --- |
| AT2G45300 | F4L23.19 | 16 | PBS A |
| AT5G02500 | HSC70-1 | 16 | PBS A |
| AT4G26640 | WRKY20 | 11 | PBS A |
| AT2G30250 | WRKY25 | 10 | PBS A |
| AT2G38470 | WRKY33 | 72 | PBS A |
| AT5G64810 | WRKY51 | 8 | PBS A |
| AT5G07440 | GDH2 | 8 | PBS B |
| AT5G56270 | WRKY2 | 4 | PBS B |
| AT5G46350 | WRKY8 | 6 | PBS B |
| AT1G50200 | ALATS | 4 | PBS C |
| AT4G34050 | CCoAOMT1 | 3 | PBS C |
| AT5G07100 | WRKY26 | 11 | PBS C |
| AT2G03340 | WRKY3 | 3 | PBS C |
| AT5G15450 | CLPB3 | 3 | PBS D |
| AT4G39980 | DHS1 | 1 | PBS D |
| AT2G17840 | ERD7 | 1 | PBS D |
| AT2G01320 | F10A8.20 | 1 | PBS D |
| AT4G31860 | F11C18.60 | 1 | PBS D |
| AT1G03370 | F15K9.2 | 3 | PBS D |
| AT5G05190 | EDR4 | 1 | PBS D |
| AT2G34420 | LHB1B2 | 1 | PBS D |
| AT1G64860 | SIGA | 11 | PBS D |
| AT4G01810 | ATSEC23A | 2 | PBS D |
| AT4G39410 | WRKY13 | 3 | PBS D |
| AT2G21900 | WRKY59 | 1 | PBS D |
| AT5G19140 | AILP1, ATAILP1 | 1 | PBS D |
| AT5G63950 | CHR24 | 10 | PBS E |

The table lists the SIB1-interacting proteins in ranked order according to the Predicted Biological Score (PBS A-E), which indicates the confidence levels of an interaction. Confidence levels were categorized as: A (very high), B (high), C (good), D (moderate), and E (highly connected node interactions).

**Table S3. List of primers used in this study**

| Primers | Sequence (5'-3') | Description |
| --- | --- | --- |
| dSpm | TACGAATAAGAGCGTCCATTTTAGAGTGA | For SM lines Transposon specific primer |
| SM_3.30601 -LP | CGATGAGAACTCGATAACCTGAC | T-DNA lines identification |
| SM_3.30601 -RP | AATCATCCCACCTATCGGAAG | T-DNA lines identification |
| SM_3.30596 -LP | CGATGAGAACTCGATAACCTGAC | T-DNA lines identification |
| SM_3.30596 -RP | AATCATCCCACCTATCGGAAG | T-DNA lines identification |
| Salk_152532c-LP | CAAAAAGGCTAAGCATGGATG | T-DNA lines identification |
| Salk_152532c-RP | TTGCTTTGAAATTCGGATCAC | T-DNA lines identification |
| Salk_021996-LP | CTTGTACGGCTGAAGCTTCAC | T-DNA lines identification |
| Salk_021996-RP | TGTAGGTGGGTGATGTTGTCC | T-DNA lines identification |
| LBb1.3 | ATTTTGCCGATTTCGGAAC | Left border primer of the T-DNA insertion, for SALK lines |
| SIB1-NYFP-F | CATGGTCCGGACTCAGATCTCGAGATGGAGTCATCATCGTCGACTTTTCTC | For BiFC assay |
| SIB1-NYFP-R | CTTGCTCACCATCAGGATCCCGGGCATAGAATCGATGCTTCCAAAGTCATT | For BiFC assay |
| Vector-NYFP-F | CCCGGGATCCTGATGGTGAGCAA | For BiFC assay |
| Vector-NYFP-R | CTCGAGATCTGAGTCCGGACCAT | For BiFC assay |
| SEC23A-CYFP-F | ATTTACGAACGATAGAGATCTCGAGATGGCTAACTTACCGAAATCGTC | For BiFC assay |
| SEC23A-CYFP-R | CACGCTGCCCAGGATCCCGGGCCTGGGCTCAGGAGGCACTA | For BiFC assay |
| Vector-CYFP-F | CCCGGGATCCTGGGCAGCGTG | For BiFC assay |
| Vector-CYFP-R | CTCGAGATCTCTATCGTTCGTAAAT | For yeast two-hybrid |
| GW-BD-SIB1-100-F | GGGGACAAGTTTGTACAAAAAAGCAGGCTTCATGGAGTCATCATCGTCGACTTTTCTC | For yeast two-hybrid |
| GW-BD-SIB1-100-R | GGGGACCACTTTGTACAAGAAAGC TGGGTGTCAAAGAACACGTGGCGCAAGATTCTCC | For yeast two-hybrid |
| GW-BD-SIB1-F | GGGGACAAGTTTGTACAAAAAAGCAGGCTTCATGGAGTCATCATCGTCGACTTTTCTC | For yeast two-hybrid |
| GW-BD-SIB1-R | GGGGACCACTTTGTACAAGAAAGC TGGGTGTCACATAGAATCGATGCTTCCAAAGTC | For yeast two-hybrid |
| GW-AD-SEC23A-F | GGGGACAAGTTTGTACAAAAAAGCAGGCTTCATGGCTAACTTACCGAAATCGTC | For yeast two-hybrid |
| GW-AD-SEC23A-R | GGGGACCACTTTGTACAAGAAAGCTGGGTGCTACCTGGGCTCAGGAGGCA | For yeast two-hybrid |
| SIB1-1-100-BD-F-ECOR1 | CGGAATTCATGGAGTCATCATCGTCGACTTTTCTC | For yeast two-hybrid |
| SIB1-1-100-BD-R-BAMH1 | CGGGATCCTCAAAGAACACGTGGCGCAAGATTCTCC | For yeast two-hybrid |
| GW-SEC23A-AD-190-421-F | GGGGACAAGTTTGTACAAAAAAGCAGGCTTCATTAGTCCTGGTCCTCAGATTATCC | For yeast two-hybrid |
| GW-SEC23A-AD-190-421-R | GGGGACCACTTTGTACAAGAAAGCTGGGTGCTATGCTCTTCTGACCACTCCC | For yeast two-hybrid |
| GW-SEC23A-AD-190-515-F | GGGGACAAGTTTGTACAAAAAAGCAGGCTTCATTAGTCCTGGTCCTCAGATTATCC | For yeast two-hybrid |
| GW-SEC23A-AD-190-515-R | GGGGACCACTTTGTACAAGAAAGCTGGGTGCTAGTCCACACCAAAGGCTTCA | For yeast two-hybrid |
| U10-SIB1-GFP-F | CTGAGTTTTTCTGATTAACAGACTAGTATGGAGTCATCATCGTCGACTTTTCTC | For generating SIB1 overexpression plant |
| U10-SIB1-GFP-R | CTTGCTCACCATGGTACCCATAGAATCGATGCTTCCAAAGTCATT | For generating SIB1 overexpression plant |
| U10-SIB1-3XFlag-F | CTGAGTTTTTCTGATTAACAGGGTACCATGGAGTCATCATCGTCGACT | For generating SIB1overexpression plant |
| U10-SIB1-3XFlag-R | CCATCATGGTCTTTGTAGTCAAGCTTCATAGAATCGATGCTTCCAAAGTC | For generating SIB1 overexpression plant |
| GW-SIB1-GFP-221-F | GGGGACAAGTTTGTACAAAAAAGCAGGCTTCATGGAGTCATCATCGTCGACTTTTCTC | For SIB1 protoplast expression |
| GW-SIB1-GFP-221-R | GGGGACCACTTTGTACAAGAAAGC TGGGTGCATAGAATCGATGCTTCCAAAGTCATT | For SIB1 protoplast expression |
| GW-SEC23A-GFP-F | GGGGACAAGTTTGTACAAAAAAGCAGGCTTCATGGCTAACTTACCGAAATCGTC | For generating SEC23A overexpression plant |
| GW-SEC23A-GFP-R | GGGGACCACTTTGTACAAGAAAGCTGGGTGCCTGGGCTCAGGAGGCACTA | For generating SEC23A overexpression plant |
| RT-AtSEC23A-F | CACATTCAGAAACTCACGAG | RT-PCR for SEC23A |
| RT-AtSEC23A-R | CCTGGGCTCAGGAGGCAC | RT-PCR for SEC23A |
| SIB1-Qpcr-F | CGTCGACTTTTCTCACCACG | Real-time quantitative PCR for SIB1 |
| SIB1-Qpcr-R | CGTCTTGGCCTGTGAGTTCT | Real-time quantitative PCR for SIB1 |
| ACTIN-2-F | AATCACAGCACTTGCACCAAGC | Real-time quantitative PCR for ACTIN2 |
| ACTIN-2-R | CCTTGGAGATCCACATCTGCTG | Real-time quantitative PCR for ACTIN2 |
| PETA-Qpcr-F | AAATCCACGAGAGGCGACTG | Real-time quantitative PCR for PETA |
| PETA-Qpcr-R | CTCCCACATTCAAAGCCCCT | Real-time quantitative PCR for PETA |
| ATPB-Qpcr-F | TCCGGTACTGGATGTAGCCT | Real-time quantitative PCR for ATPB |
| ATPB-Qpcr-R | CGCCGACTGGAACACTTAGA | Real-time quantitative PCR for ATPB |
| ATPI- Qpcr-F | CCAGGTCCATGCCCAAGTTC | Real-time quantitative PCR for ATPI |
| ATPI- Qpcr-R | GGTAAAAGGGCTCCTGACCA | Real-time quantitative PCR for ATPI |
| nSIB1-gus-zeo-F | GGGGACAAGTTTGTACAAAAAAGCAGGCTTCAAGAATGAAAAGGATTCGAGTTTGA | For generating *SIB1 GUS* plant |
| nSIB1-gus-zeo-R | GGGGACCACTTTGTACAAGAAAGCTGGGTGGTTCGGTTCCTGTAAGAGAATTCAG | For generating *SIB1 GUS* plant |
| PR1-Qpcr-F | ACGGGGAAAACTTAGCCTGG | Real-time quantitative PCR for PR1 |
| PR1-Qpcr-R | TTGGCACATCCGAGTCTCAC | Real-time quantitative PCR for PR1 |
| FRK1-Qpcr-F | TTAGTTTCAGACCGCCCGAC | Real-time quantitative PCR for FRK1 |
| FRK1-Qpcr-R | ATGGTAATTCCGCTCACGGG | Real-time quantitative PCR for FRK1 |
| GW--SIB1-YC-F | GGGGACAAGTTTGTACAAAAAAGCAGGCTTCATGGAGTCATCATCGTCGACTTTTCTC | For BiFC assay in *N. benthamiana* |
| GW-SIB1-YC-R | GGGGACCACTTTGTACAAGAAAGCTGGGTGCATAGAATCGATGCTTCCAAAGTCATT | For BiFC assay in *N. benthamiana* |
| GW--SEC23A-YN-F | GGGGACAAGTTTGTACAAAAAAGCAGGCTTCATGGCTAACTTACCGAAATCGTC | For BiFC assay in *N. benthamiana* |
| GW-SEC23A-YN-R | GGGGACCACTTTGTACAAGAAAGCTGGGTGCCTGGGCTCAGGAGGCACTA | For BiFC assay in *N. benthamiana* |
| GW-SEC23D-YN-F | GGGGACAAGTTTGTACAAAAAAGCAGGCTTCATGGCAGTGAGAGCAACGGTG | For BiFC assay in *N. benthamiana* |
| GW-SEC23D-YN-R | GGGGACCACTTTGTACAAGAAAGCTGGGTGCTTCATGTATTCAGTGACACGCTG | For BiFC assay in *N. benthamiana* |
